# Chinese Medicinal Formula (MHGWT) for Relieving Diabetic Neuropathic Pain: A Randomized, Double-Blind, Placebo-Controlled Trial

**DOI:** 10.1155/2013/767498

**Published:** 2013-08-25

**Authors:** Chia-I Tsai, Tsai-Chung Li, Ming-Hong Chang, Shih-Yi Lin, I-Te Lee, Cheng-Hung Lee, Tzu-Yuan Wang, Yi-Chang Su

**Affiliations:** ^1^Graduate Institute of Chinese Medicine, College of Chinese Medicine, China Medical University, Taichung 40402, Taiwan; ^2^Department of Traditional Chinese Medicine, Taichung Veterans General Hospital, Taichung 40705, Taiwan; ^3^School of Chinese Medicine, College of Chinese Medicine, China Medical University, Taichung 40402, Taiwan; ^4^Graduate Institute of Biostatistics, China Medical University, Taichung 40402, Taiwan; ^5^Department of Healthcare Administration, College of Health Science, Asia University, Taichung 41354, Taiwan; ^6^Section of Neurology, Taichung Veterans General Hospital, Taichung 40705, Taiwan; ^7^Division of Endocrinology and Metabolism, Department of Internal Medicine, Taichung Veterans General Hospital, Taichung 40705, Taiwan; ^8^Institute of Medicine, Chung Shan Medical University, Taichung 40201, Taiwan; ^9^School of Medicine, National Yang-Ming University, Taipei 11221, Taiwan; ^10^Division of Endocrinology and Metabolism, Department of Internal Medicine, China Medical University Hospital, Taichung 40447, Taiwan

## Abstract

*Objective*. To investigate the effects of modified *Hungqi Guizhi Wuwu Tang* (MHGWT), a formula that comprises Chinese medicinal herbs, in relieving neuropathic pain in diabetics. *Method*. Between March 2008 and April 2009, 112 participants were randomly assigned to either the MHGWT group, whose members received MHGWT (*n* = 56), or the control group, whose members received a placebo (*n* = 56). Diabetic neuropathic pain (DNP) was rated using the 15-item Short-Form Brief Pain Inventory (SF-BPI), the 17-item Short-Form McGill Pain Questionnaire (SF-MPQ), the 13-item Modified Michigan Neuropathy Screening Instrument (MMNSI), and the 36-item “SF-36.” Nerve conduction studies (NCSs) were performed before and after treatment. *Results*. After 12 weeks of treatment, the SF-MPQ and SF-BPI scores of the MHGWT group were significantly (*P* < 0.05) reduced and a significant difference between the groups was observed (*P* < 0.05). The levels of NCS in the MHGWT group were nonsignificantly (*P* > 0.05) reduced, and no significant difference in NCS level was observed between the groups (*P* > 0.05). *Conclusions*. MHGWT shows promise in relieving DNP and deserves further investigation.

## 1. Introduction

Neuropathy is the most common complication associated with diabetes, and sensorimotor diabetic peripheral neuropathy (DPN) is the most common form of diabetic neuropathy [1, 2]. It is characterized by a progressive loss of nerve fibers that predisposes the sufferer to painful or numb extremities, ulceration, and amputation and results in a large disease burden in terms of inability to work, significantly reduced quality of life, and consumption of healthcare resources [3, 4]. In Taiwan, the prevalence of DPN in patients with diabetes mellitus is approximately 20–30% [5].

Many agents are currently utilized to manage diabetic neuropathic pain but only two, pregabalin and duloxetine, have been approved by the American FDA for this indication [6]. However, the significant side effects of these agents, including dizziness, somnolence, infection, peripheral edema [7], hepatotoxicity, and related deterioration of blood cells, are not trivial [8]. Consequently, they must be used with caution [9]. For thousands of years, the Chinese herbal medicine *Hungqi Guizhi Wuwu Tang *(HGWT) has been prescribed to improve the circulation of the extremities [10]. Clinical investigations have suggested that it has therapeutic potential for DPN [11], with the added advantages of lower cost and fewer side effects compared with the aforementioned agents [12]. Several clinical trials have been conducted, but most of them were of low methodological quality. Thus, any positive findings concerning the efficacy of Chinese herbal medicines for treating DPN should be treated with caution [13]. In order to reliably evaluate the effectiveness of HGWT in treating DPN, high-quality clinical trials are required.

## 2. Materials and Methods

### 2.1. Participants

The participants in this study were men and women aged over 18 years with a Michigan Neuropathy screening score of at least 3. They were recruited between March 2008 and April 2009 from the outpatient clinic of the Taichung Veterans General Hospital, Taichung, Taiwan. Participants had to have been diagnosed with DM based on criteria consistent with those recommended by the American Diabetes Association (ADA) and were required to have fasting plasma ≥126 mg/dL, or two-hour plasma glucose ≥200 mg/dL as measured by the oral glucose tolerance test (OGTT) on two separate occasions, or random plasma glucose ≥200 mg/dL with symptoms (polyuria, polydipsia, and unexplained weight loss) [14]. Women who were pregnant or breastfeeding were excluded from the study, as were patients who satisfied any of the following conditions: renal dysfunction (serum creatinine (Cr) > 1.3 mg/dL); active liver disease or hepatic dysfunction (glutamic pyruvic transaminase (GPT) > double the upper limit of the normal range (ULN)); and cerebrovascular disease, previous cardiovascular surgery, myocardial infarction, or major trauma or operations up to six months prior to the study period, or participation in another clinical trial within 30 days before consideration for entry into this study. Patients who were taking analgesics (such as NSAIDs and tramadol), antidepressants (such as TCAs, SSRIs, and duloxetine), capsaicin topical cream, and anticonvulsants (such as pregabalin) or Chinese medicinal herbs were considered for screening only after a four-week washout period. All the enrolled participants were able to communicate well enough to complete the questionnaires and were capable of responding to the nerve conduction study (NCS). The participants were excluded from the study if they had any history of psychiatric disorders, history of alcohol or drug abuse, and any condition associated with poor compliance with medical treatment. The study complied with the Declaration of Helsinki. The relevant institutional ethics review boards approved the protocol, and all participants provided informed consent. This trial was registered with ClinicalTrials.gov, number NCT00886655.

### 2.2. Randomization and Estimation of Sample Size

A permuted-block randomization was utilized to assign subjects randomly, based on the order of entry into two treatment groups. Each group received either MHGWT or a placebo. A sample size of 112 patients was used to evaluate and compare the efficacies and safeties of MHGWT and the placebo. The sample size was chosen according to the primary efficacy outcome. The change from the baseline mean Short-Form Brief Pain Inventory (SF-BPI) score to that after 12 weeks was determined for the two groups. The two-sided alpha (type I error) was set to 0.05 and the beta (type II error) was set to 0.10 (power of 90%). The effect size was 0.5 and the standard deviation (SD) of the BPI scores was 0.5. Based on these assumptions, a sample size of approximately 29 subjects per group was required for an assumed 30% loss of follow-up.

### 2.3. Interventions

The mixture of Chinese medicinal herbs that is known as modified *Hungqi Guizhi Wuwu Tang* (MHGWT) is composed of the following seven species: *Astragali Radix *(Leguminosae), radix; *Cinnamomum cassia* Presl., ramulus; *Paeonia lactiflora* Pall., radix; *Zingiber officinale *(Willd.) Rosc., radix; *Ziziphus jujuba *Mill.,fructus; *Spatholobus suberectus* Dunn., caulis; and *Pheretima aspergillum* (Perrier). The Chinese medicinal herb formula, MHGWT, given to the treatment group was powdered and packed in aluminum foil according to Good Manufacturing Practices (GMP) by Kaiser Pharmaceuticals Co., Ltd., Tainan, Taiwan. The placebo was composed of the same medicine that was administered to the MHGWT group but at one-tenth the concentration. Identical packets of MHGWT and placebo powder, which were dispensed by the hospital pharmacy, were marked with codes that corresponded to the participants' names. Participants were asked to take one pack (4 g MHGWT or placebo) three times daily, 30 min after breakfast, lunch, and dinner, throughout the 84 days of the study and to return the remnants so that the packs could be counted at each visit to the clinic. High-performance liquid chromatography (HPLC) fingerprints were obtained to identify substances in the mixtures and to ensure that the product was of consistent quality. Contamination screening for heavy metals such as lead, arsenic, cadmium, and mercury was performed to ensure safety for human consumption. The total concentration of heavy metals was less than 100 ppm. Please refer to Figure 2 for report of test of Chinese medicinal herbs for heavy metals.

### 2.4. Protocol

**Figure 1:**
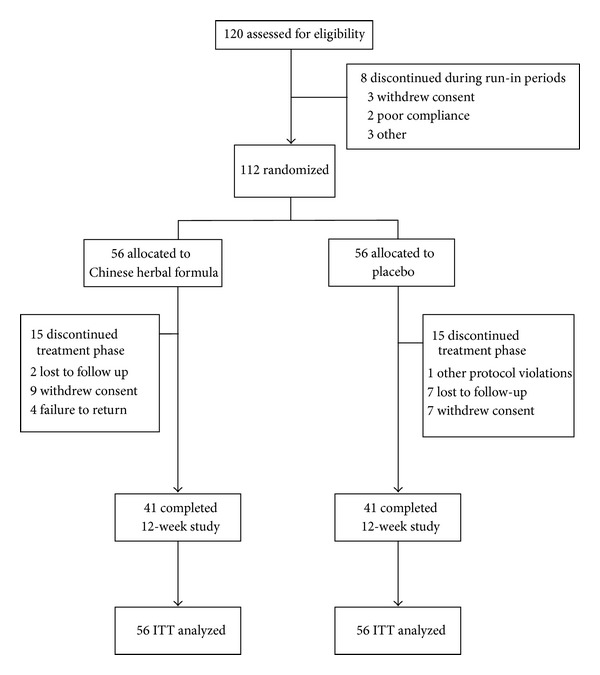
Participant flowchart depicting the randomization, treatment, and followup in MHGWT and placebo groups.

**Figure 2:**
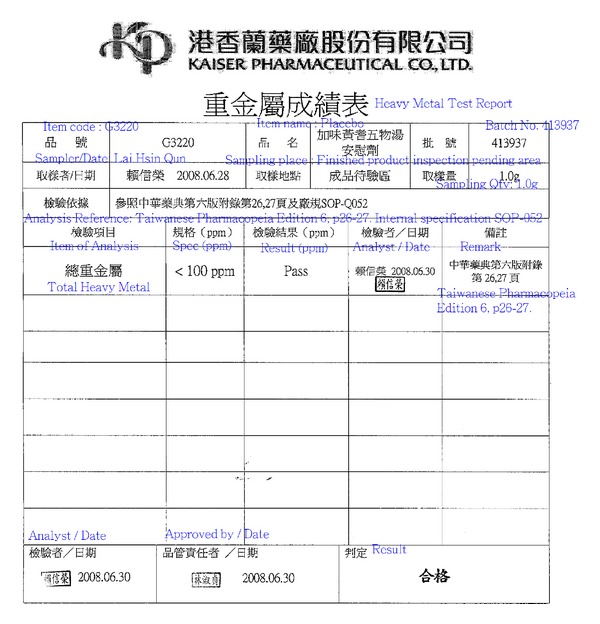
Report of test of Chinese medicinal herbs for heavy metals.

After a minimum run-in period of four weeks with stable plasma glucose, 112 patients were randomly assigned to 12 weeks of treatment with powdered placebo or MHGWT (Figure 1). The study was double-blind. Participants were reviewed every four weeks and blood samples were obtained after fasting overnight for 12 hours. The laboratory staff responsible for the analyses were blind to the treatment and received samples that were labeled with only name codes and dates.

### 2.5. Outcome Measures

#### 2.5.1. Short-Form Brief Pain Inventory (SF-BPI)

The SF-BPI questionnaire is self-administered by the patient and comprises 15 questions concerning various aspects of pain, which were adopted from the standard BPI. Questions 1 and 2 concern the type and location of the pain. Questions 3 to 6 concern the degree of the worst, mildest, and average pain during the preceding week on a 0–10 scale. Questions 7 and 8 are related to treatment of the pain and the percentage by which the treatment reduced the pain. Questions 9–15 assess the extent to which the pain affects general activity, mood, ability to walk, normal work, relationships, sleep, and enjoyment of life, on a scale of 0–10. A higher SF-BPI score indicates more severe neuropathic symptoms. Cronbach's *α* for this study ranged from 0.83 to 0.99.

#### 2.5.2. Short-Form McGill Pain Questionnaire (SF-MPQ)

The SF-MPQ comprises a series of questions regarding various aspects of the reported pain, with a total of 15 descriptors (11 sensory, four affective). It also includes the Present Pain Intensity (PPI) index (a simple verbal description of pain) and a Visual Analogue scale (VAS) (a quantitative measure of pain), both of which were adopted from the standard MPQ. A higher SF-MPQ score indicates more severe neuropathic symptoms. Cronbach's *α* ranged from 0.70 to 0.89.

#### 2.5.3. Modified Michigan Neuropathy Screening Instrument (MMNSI)

The MMNSI questionnaire comprises 13 self-administered questions concerning sensation in the foot, addressing positive (burning, tingling) and negative (numbness, temperature sensitivity) sensory symptoms, cramps, and muscle weakness. A higher MMNSI score indicates more severe neuropathic symptoms. For each question, answers are transformed to a scale from zero (no symptom) to 10 (the severest possible symptom). The instrument has been validated to evaluate symptoms of diabetic neuropathy. Cronbach's *α* coefficient ranged from 0.78 to 0.91.

#### 2.5.4. SF-36

The SF-36 is a short questionnaire with 36 questions measuring eight multiitem variables: physical functioning (PF, 10 items), social functioning (SF, two items), role limitations due to physical problems (RP, four items), role limitations due to emotional problems (RE, three items), mental health (MH, five items), vitality (VT, four items), pain (BP, two items), and general perception of health (GH, five items). For each variable, scores are coded, summed, and transformed to a scale from 0 (the worst possible health state) to 100 (the best possible health state). The scores on the SF-36 Physical Component Summary (PCS) and the Mental Component Summary (MCS) scales are derived using the standard SF-36 scoring algorithms. A higher SF-36 score indicates better health. Cronbach's *α* ranged from 0.68 to 0.90, except for SF and VT.

#### 2.5.5. Nerve Conduction Study (NCS)

The NCS provides an objective estimate of the functioning of the peripheral nerve. It uses a stimulator, grounding electrode, reference electrode, and recording electrode. The routine NCS in our laboratory included tibial and peroneal nerves (motor function) and sural nerve (sensory function). The differences between the pre- and posttherapy electrophysiologic measurements of distal motor and sensory latencies (DML, DSL), amplitudes of the compound muscle action potentials (CMAPs) and sensory nerve action potentials (SNAPs), motor nerve conduction velocities (MNCVs), and F wave latencies were recorded using an electromyelogram (Viking Select, Nicolet, USA).

#### 2.5.6. Safety Analysis

Safety was evaluated by measuring serum GPT and Cr levels in all patients who had taken at least one dose of medication. All adverse effects that were observed during the clinical trial were recorded. The investigator studied the probability of their relationship to the study drug (definitely, probably, possibly, unlikely, and definitely not) and their intensity (mild, moderate, and severe). Physical examinations and clinical laboratory determinations were performed upon screening, randomization, and study termination.

### 2.6. Statistical Analysis

Descriptive statistics, including observations, means, standard deviations, and percentages, were used to summarize the baseline variables. All tests were two sided and were performed using the 0.05 level of significance. The demographic information about the two groups was obtained using an independent *t*-test for continuous variables and a chi-square test for categorical variables. Changes from the baseline of scores in the questionnaire, safety parameters, the results of laboratory examinations, and the results of nerve conduction studies were analyzed based on the intention-to-treat principle, and the independent *t*-test was performed to determine between-group variation. The repeated ANOVA was assessed using Scheffe's test as a post hoc comparison. Fisher's exact test was performed to compare the numbers of subjects with adverse effects in the two groups.

## 3. Results 

### 3.1. Study Population

Of the 120 patients screened, eight were ineligible. The main reasons for ineligibility included violation of selection criteria at entry (*n* = 3), withdrawal of consent (*n* = 3), and poor compliance (*n* = 2). A total of 82 (73%) of the 112 recruited subjects completed the 12-week study without any notable protocol violation. The reasons for the 30 withdrawals were withdrawal of consent (*n* = 16), absence during follow-up (*n* = 9), failure to return (*n* = 4), and deviation from protocol (*n* = 1) (Figure 1). The demographic and clinical characteristics of the study subjects did not differ significantly between the MHGWT and placebo groups (Table 1). The mean age of the patients that underwent the MHGWT treatment was 60.46 years (standard deviation, SD = 10.60 years) and was 60.71 years (SD = 10.20 years) in the placebo group. The baseline demographic and biomarker characteristics of the two groups were well balanced (Table 1). 

### 3.2. Efficacy Analysis

The reductions in mean SF-BPI scores in the sensory and daily life domains in the MHGWT group exceeded those observed in the control group during all treatment phases, and the differences were the largest during weeks 1–12 (−15.64 ± 8.78 versus 0.53 ± 7.88, *P* <  0.001) (−15.64 ± 8.78 versus 0.53 ± 7.88, *P* < 0.001) (Table 2).

The total (sensory plus affective) SF-MPQ scores of the MHGWT group were significantly lower than those of the control group during weeks 1–4 (−2.36 ± 3.62 versus 0.47 ± 3.65, *P* < 0.001), weeks 1–8 (−4.29 ± 3.80 versus 0.39 ± 3.45, *P* < 0.001), and weeks 1–12 (−4.93 ± 4.27 versus 0.34 ± 4.08, *P* < 0.001). Significant differences from the baseline in the VAS and PPI domains during weeks 4, 8, and 12 were observed for both groups, as presented in Table 3 (*P* < 0.001).

Table 4 compares the baseline Modified Michigan Neuropathy Screening Instrument (MMNSI) scores of the MHGWT and placebo groups as well as the changes in scores compared with those at time points 2, 3, and 4. The mean decrease in the MMNSI score of the MHGWT group exceeded that of the placebo group during weeks 1–4 (−11.75 ± 10.69 versus −1.02 ± 9.01, *P* < 0.001), weeks 1–8 (−23.05 ± 13.58 versus −2.97 ± 11.65, *P* < 0.001), and weeks 1–12 (−28.05 ± 14.65 versus −1.03 ± 11.42, *P* < 0.001). The improvement in MMNSI scores differed significantly between the two groups after 4, 8, and 12 weeks of treatment, as presented in Table 4 (*P* < 0.001).

After the first eight weeks of treatment with MHGWT, the short-form 36 questionnaire scores in the two groups differed significantly for role limitation due to physical problems (RP), bodily pain (BP), general perception of health (GH), social functioning (SF), role limitation due to emotional problems (RE), and physical component scale (PCS), as shown in Table 5 (*P* < 0.01). After 12 weeks of treatment with MHGWT, the two groups' short-form 36 questionnaire scores differed significantly from their baseline values for all domains, as shown in Table 5 (*P* < 0.01).

Neither group exhibited significant changes on the NCS, except for the F-wave in the peroneal nerve (Table 6).

The repeated ANOVA was assessed using Scheffe's test and revealed that all domains of SF-BPI, Short-Form McGill Pain Questionnaire (SF-MPQ), and Modified Michigan Neuropathy Screening Instrument (MMNSI) differed significantly from their respective baseline values for the two groups, except for the affective domain of SF-MPQ after 4 and 8 weeks of treatment, as shown in Tables 2–4 (*P* < 0.01). All domains of SF-36 differed significantly from their baseline values in the two groups, except for the domains of vitality, role limitation due to emotional problems, mental health, and mental component scale after 8 weeks of treatment, as shown in Table 5 (*P* < 0.01).

### 3.3. Safety Issues

There were 39 events of dry mouth, 24 events of constipation, and one event of bitter sensation in the mouth that was judged to be probably related to the treatment, but the magnitudes of these effects were relatively small, and no major adverse event occurred (Table 7).

## 4. Discussion

In this randomized, double-blind, and placebo-controlled trial, a Chinese medicinal formula, modified *Hungqi Guizhi Wuwu Tang* (MHGWT), was found to be effective and well tolerated in diabetic patients with DPN. The MHGWT regimen reduced the pain and numbness of extremities and improved quality of life during the 12 weeks of treatment. Subjective and objective tests were performed and significant outcomes were detected using well-validated questionnaires and NCS during the study period. 

With respect to the safety of MHGWT, no deterioration of hepatic and renal functions or major adverse event was detected throughout the period of treatment. This finding is consistent with our experience of practicing traditional Chinese medicine (TCM), as indicated in Table 1. Since pain is a subjective symptom, three pain questionnaires were utilized in this investigation to evaluate different aspects of the burden that was imposed by DPN. The well-validated SF-36 was also utilized to explore the impact on quality of life. NCSs were used to make an objective measurement. The significant reduction of MMNSI scores indicated that treatment with MHGWT had an effect within four weeks. The declines in the MMNSI scores increased with the period of treatment. The consistent declines in scores in the responses of the patients with DPN to the three pain questionnaires in this study imply that the efficacy of MHGWT is reliable. This trend was also revealed by the SF-36 scores for quality of life. 

In the current study, in addition to investigating various health benefits by subjective questionnaires, we attempted to show whether objective parameters, such as electrophysiological values, changed in response to treatment. Pain is transmitted through small or unmyelinated fibers, but routine NCS is used to investigate large myelinated fibers. Therefore, the absence of a significant improvement in NCS between pre- and posttherapy phases is reasonable. Although we failed to demonstrate objective evidence of improvement in pain in the experimental group, it is important to note the limitations of current techniques for reliable clinical assessment of pain and to note that self-reporting is widely considered to be a more effective measure of pain in clinical practice.

*Hungqi Guizhi Wuwu Tang* is a classic formula used in traditional Chinese medicine for improving microcirculation. To improve its efficacy, *Spatholobus suberectus* Dunn., caulis and *Pheretima aspergillum* (Perrier) were added. A number of studies have shown evidence of the efficacy of MHGWT in improving neuropathy [15–18]. However, very few clinical trials of high quality have been performed to investigate treatment of DPN with Chinese herbal medicine [19]. Such studies did not employ standardized treatment, well-validated questionnaires, adequate blindness or randomization, or the Good Clinical Practice (GCP) protocol [12]. The use of MHGWT warrants further study as it has fewer side effects and is safer than conventional drugs. Because the risk of developing neuropathic pain increases with age, it may provide an alternative therapy for the elderly patients [20].

The present study has some limitations. First, the success of blinding was not tested. Second, the poor ability of the subjects due to factors that often accompany old age, such as degeneration, use of multiple medications, and chronic illness, may have negatively affected the reliability of the responses to the self-administered questionnaires. Third, the main population in the study was older adults. Therefore, the effect of MHGWT on younger patients with DPN remains to be investigated.

Several studies on the possible mechanisms of ingredients of MHGWT that improve DPN have been conducted. The mechanism of endoneurial hypoxia resulting from arteriovenous shunting with the proliferation of new leaky neural vessels has been reported [21]. Previous studies suggest that the therapeutic mechanism of MHGWT may involve improvement of the circulation supplying peripheral nerves [22]. Inflammation contributes to the development of diabetes; *Astragalus membranaceus* (*Hungqi*) play an important role in lowering blood glucose and controlling inflammation through AMPK activity [23–27]. Recent studies have shown Astragaloside IV to be an aldose-reductase inhibitor and a free-radical scavenger and it exerts protective effects against the progression of peripheral neuropathy in STZ-induced diabetes in rats [28, 29]. Astragalus polysaccharides (APS) administration could also prevent the development of lipotoxicity through a mechanism dependent on the PPAR*α*-mediated regulatory pathways [30]. *Paeonia lactiflora* (*Baishao*) helps to increase the antioxidant capacity of an organism and protect it against lipid peroxidation induced by oxidative stress [31, 32]. As TCM targets the underlying disturbed homeostasis, studies concerning the mechanisms related to the characteristics of TCM syndrome differentiation or body constitution that reflect the inner health status of the body should be conducted too [33]. 

## 5. Conclusions

MHGWT appears to be a well-tolerated and effective therapeutic alternative for treating painful sensation in patients, especially older patients, with DPN.

## Figures and Tables

**Table 1:** Patient's assignment, values at screening, and safety values.

Variables	Group, number		*P* value
	MHGWT group (*n* = 56)	Placebo group (*n* = 56)	
Screening values			
Male, *n* (%)	29 (51.79)	28 (50)	1.00
Female, *n* (%)	27 (48.21)	28 (50)	1.00
Age (years)	60.71 ± 10.20	60.46 ± 10.60	0.90
Height (cm)	162.14 ± 7.04	162.11 ± 8.07	0.98
Weight (kg)	67.00 ± 12.44	66.49 ± 10.28	0.81
Body-mass index	25.41 ± 3.87	25.24 ± 3.08	0.80
Duration of diabetes (years)	10.59 ± 6.49	9.91 ± 6.68	0.59
Duration of DPN (years)	2.49 ± 1.86	9.91 ± 6.68	0.35
Baseline safety values			
GPT (u/L)	25.82 ± 13.67	23.04 ± 8.24	0.20
Creatinine (mg/dL)	1.06 ± 0.25	1.06 ± 0.22	0.88
Fasting glucose (mmol/L)	155.84 ± 50.70	154.68 ± 47.82	0.90
HbA1c (%)	8.11 ± 1.71	7.86 ± 1.63	0.42
Triglycerides (mmol/L)	171.69 ± 195.91	200.76 ± 183.67	0.42
Total cholesterol (mmol/L)	189.65 ± 38.08	187.18 ± 49.22	0.77

Screening values and safety values were obtained at baseline.

Data were presented as mean ± SD.

*P* values are calculated for the comparison of difference between MHGWT and placebo groups by independent *t*-test and chi-square test.

GPT: glutamic pyruvic transaminase.

HbA1c: glycosylated hemoglobin.

**Table 2:** Changes of scores of Short-Form Brief Pain Inventory questionnaire after interventions.

Domains	Group, number		*P* value
	MHGWT group (*n* = 56)	Placebo group (*n* = 56)	
Sensory			
Baseline	25.43 ± 8.13	21.45 ± 8.37	0.01*
Time 2 − baseline	− 5.48 ± 6.70	0.20 ± 8.90	0.001***
Time 3 − baseline	− 12.62 ± 7.57	0.16 ± 8.19	<0.0001***
Time 4 − baseline	− 15.64 ± 8.78	0.53 ± 7.88	<0.0001***
Scheffe's test for time effect	a > b > c > d		
Daily life			
Baseline	27.43 ± 12.25	23.34 ± 13.00	0.10
Time 2 − baseline	− 9.77 ± 9.10	0.32 ± 11.25	<0.0001***
Time 3 − baseline	− 15.14 ± 10.63	− 3.47 ± 13.84	<0.0001***
Time 4 − baseline	− 18.36 ± 11.66	− 4.66 ± 14.49	<0.0001***
Scheffe's test for time effect	a > b > c, d	a > d	

Time 2 was in the end of 4 weeks, time 3 was in the end of 8 weeks, and time 4 was in the end of 12 weeks.

Data were presented as mean ± SD.

*P* values are calculated for the comparison of difference between MHGWT and placebo groups by independent *t*-test.

*P < 0.05; ***P < 0.001.

a: time 1; b: time 2; c: time 3; d: time 4.

**Table 3:** Changes of scores of Short-Form McGill Pain Questionnaire after interventions.

Domains	Group, number		*P* value
	MHGWT group (*n* = 56)	Placebo group (*n* = 56)	
Sensory			
Baseline	7.30 ± 3.42	5.91 ± 3.77	0.04*
Time 2 − baseline	−1.93 ± 2.58	0.04 ± 3.04	0.001**
Time 3 − baseline	−3.36 ± 3.01	0.13 ± 3.06	<0.001***
Time 4 − baseline	−3.85 ± 3.20	−0.07 ± 3.36	<0.001***
Scheffe's test for time effect	a > b > c, d		
Affective			
Baseline	2.22 ± 1.54	1.66 ± 1.75	0.08
Time 2 − baseline	−0.43 ± 1.52	0.43 ± 1.06	0.003**
Time 3 − baseline	−0.93 ± 1.60	0.26 ± 1.13	<0.001***
Time 4 − baseline	−1.07 ± 1.68	0.41 ± 1.26	<0.001***
Scheffe's test for time effect	a > b, c, d; b > d		
VAS			
Baseline	4.98 ± 1.39	4.07 ± 1.53	0.002**
Time 2 − baseline	−1.55 ± 1.16	−0.04 ± 1.38	0.002**
Time 3 − baseline	−2.50 ± 1.61	−0.10 ± 1.56	<0.001***
Time 4 − baseline	−3.10 ± 1.69	−0.31 ± 1.61	<0.001***
Scheffe's test for time effect	a > b > c > d		
PPI			
Baseline	2.52 ± 0.72	2.18 ± 0.69	0.01**
Time 2 − baseline	−0.45 ± 0.70	0.09 ± 0.75	<0.001***
Time 3 − baseline	−0.98 ± 0.72	−0.08 ± 0.78	<0.001***
Time 4 − baseline	−1.20 ± 0.75	−0.17 ± 0.89	<0.001***
Scheffe's test for time effect	a > b > c, d		
Total			
Baseline	9.52 ± 4.17	7.57 ± 5.09	0.03*
Time 2 − baseline	−2.36 ± 3.62	0.47 ± 3.65	<0.001***
Time 3 − baseline	−4.29 ± 3.80	0.39 ± 3.45	<0.001***
Time 4 − baseline	−4.93 ± 4.27	0.34 ± 4.08	<0.001***
Scheffe's test for time effect	a > b > c, d		

Time 2 was in the end of 4 weeks, time 3 was in the end of 8 weeks, and time 4 was in the end of 12 weeks.

Data were presented as mean ± SD.

*P* values are calculated for the comparison between MHGWT and placebo groups by independent *t*-test.

*P < 0.05; **P < 0.01; ***P < 0.001.

a: time 1; b: time 2; c: time 3; d: time 4.

**Table 4:** Changes of scores of Modified Michigan Neuropathy Screening Instrument after interventions.

Domains	Group, number		*P* value
	MHGWT group (*n* = 56)	Placebo group (*n* = 56)	
Baseline	45.02 ± 17.07	32.95 ± 14.38	<0.001***
Time 2 − baseline	−11.75 ± 10.69	−1.02 ± 9.01	<0.001***
Time 3 − baseline	−23.05 ± 13.58	−2.97 ± 11.65	<0.001***
Time 4 − baseline	−28.05 ± 14.65	−1.03 ± 11.42	<0.001***
Scheffe's test for time effect	a > b > c, d		

Time 2 was in the end of 4 weeks, time 3 was in the end of 8 weeks, and time 4 was in the end of 12 weeks.

Data were presented as mean ± SD.

*P* values are calculated for the comparison between MHGWT and placebo groups by independent *t*-test.

***P < 0.001.

a: time 1; b: time 2; c: time 3; d: time 4.

**Table 5:** Changes of scores of short-form 36 questionnaire after interventions.

Domains	Group, number		*P* value
	MHGWT group (*n* = 56)	Placebo group (*n* = 56)	
Physical function			
Time 1^a^	68.16 ± 22.05	64.91 ± 21.38	0.43
Time 2^b^	73.80 ± 21.14	66.12 ± 24.39	0.11
Time 3^c^	81.70 ± 17.52	73.07 ± 20.32	0.04*
Time 4^d^	83.80 ± 14.38	69.38 ± 21.68	<0.001***
Scheffe's test for time effect	a, b < c, d	a, b < c	
Role limitation due to physical problems			
Time 1^a^	25.00 ± 33.74	26.82 ± 38.75	0.79
Time 2^b^	36.41 ± 31.93	21.94 ± 34.47	0.04*
Time 3^c^	61.36 ± 34.71	32.95 ± 38.04	<0.001***
Time 4^d^	73.91 ± 32.90	28.13 ± 37.05	<0.001***
Scheffe's test for time effect	a, b < c, d		
Bodily pain			
Time 1^a^	47.09 ± 10.95	50.13 ± 17.53	0.28
Time 2^b^	57.67 ± 11.61	54.02 ± 15.78	0.20
Time 3^c^	64.39 ± 14.47	56.05 ± 15.61	0.01*
Time 4^d^	66.07 ± 14.15	57.10 ± 15.19	0.004**
Scheffe's test for time effect	a < b < c, d	a < c, d	
General perception of health			
Time 1^a^	27.65 ± 17.18	31.89 ± 17.02	0.19
Time 2^b^	39.04 ± 17.37	33.14 ± 18.59	0.11
Time 3^c^	48.98 ± 18.70	34.64 ± 18.83	<0.001***
Time 4^d^	51.59 ± 19.67	33.15 ± 19.02	<0.001***
Scheffe's test for time effect	a < b < c, d		
Vitality			
Time 1^a^	46.23 ± 13.37	50.82 ± 18.25	0.13
Time 2^b^	51.20 ± 12.16	50.51 ± 13.63	0.80
Time 3^c^	56.02 ± 13.06	52.50 ± 13.91	0.22
Time 4^d^	58.26 ± 11.70	51.46 ± 14.25	0.01*
Scheffe's test for time effect	a, b < d; a < c		
Social functioning			
Time 1^a^	64.25 ± 17.11	65.68 ± 17.05	0.66
Time 2^b^	69.29 ± 16.61	66.07 ± 18.04	0.37
Time 3^c^	80.40 ± 17.13	70.17 ± 17.72	0.007**
Time 4^d^	80.16 ± 19.29	69.53 ± 16.08	0.005**
Scheffe's test for time effect	a, b < c, d		
Role limitation due to emotional problems			
Time 1^a^	49.12 ± 41.84	52.12 ± 42.92	0.71
Time 2^b^	61.48 ± 42.02	42.86 ± 41.39	0.03*
Time 3^c^	75.00 ± 34.57	51.52 ± 40.95	0.005**
Time 4^d^	82.61 ± 33.51	40.97 ± 37.81	<0.001***
Scheffe's test for time effect	a, b < d; a < c		
Mental health			
Time 1^a^	63.02 ± 14.53	64.95 ± 16.63	0.52
Time 2^b^	69.57 ± 13.19	66.20 ± 12.28	0.20
Time 3^c^	71.82 ± 15.14	67.36 ± 12.65	0.14
Time 4^d^	73.39 ± 13.18	66.17 ± 13.25	0.009**
Scheffe's test for time effect	a < b, c, d		
Physical component scale			
Time 1^a^	34.97 ± 7.02	35.14 ± 7.63	0.90
Time 2^b^	38.41 ± 7.14	35.91 ± 9.03	0.14
Time 3^c^	43.72 ± 7.84	38.17 ± 8.54	0.002**
Time 4^d^	45.36 ± 7.05	37.69 ± 8.50	<0.001***
Scheffe's test for time effect	a < b < c, d	a < c, d	
Mental component scale			
Time 1^a^	43.80 ± 9.51	45.51 ± 9.66	0.35
Time 2^b^	47.11 ± 8.50	44.53 ± 7.96	0.13
Time 3^c^	49.05 ± 9.29	45.50 ± 7.81	0.06
Time 4^d^	49.94 ± 8.77	44.11 ± 7.26	<0.001***
Scheffe's test for time effect	a < c, d		

Time 2 was in the end of 4 weeks, time 3 was in the end of 8 weeks, and time 4 was in the end of 12 weeks.

Data were presented as mean ± SD.

*P* values are calculated for the comparison between MHGWT and placebo groups by independent *t*-test.

*P* < 0.05*; *P* < 0.01**; *P* < 0.001***.

a: time 1; b: time 2; c: time 3; d: time 4.

**Table 6:** Summary of electrophysiology before and after interventions.

Variables	Group, number		P value
	MHGWT group (n = 56)	Placebo group (n = 56)	
*Peroneal nerve *			
Distal latency (ms)			
Pretherapy	4.44 ± 0.83	4.32 ± 0.95	0.48
Posttherapy	4.80 ± 0.99	4.43 ± 0.97	0.14
MNCV (m/s)			
Pretherapy	42.92 ± 6.64	42.27 ± 5.69	0.59
Posttherapy	40.35 ± 9.63	54.06 ± 71.27	0.27
Amplitude (*μ*V)			
Pretherapy	3479.09 ± 2552.01	3783.02 ± 2122.90	0.50
Posttherapy	3436.67 ± 3021.24	3631.43 ± 2277.87	0.77
F-wave (ms)			
Pretherapy	47.74 ± 8.06	50.04 ± 5.42	0.11
Posttherapy	46.83 ± 8.60	51.25 ± 6.46	0.03*
*Sural nerve *			
Amplitude (*μ*V)			
Pretherapy	10.09 ± 5.12	9.54 ± 5.43	0.66
Posttherapy	10.61 ± 6.61	10.99 ± 6.62	0.85
Distal latency (ms)			
Pretherapy	2.87 ± 0.41	2.84 ± 0.39	0.75
Posttherapy	2.91 ± 0.48	2.90 ± 0.27	0.94

Data were presented as mean ± SD.

*P* values are calculated for the comparison between MHGWT and placebo groups by independent *t*-test.

*P < 0.05.

**Table 7:** Comparison of adverse events.

Adverse events	Group, number				*P* value
	MHGWT group (*n* = 56)		Placebo group (*n* = 56)	
	No.	%	No.	%
Dry mouth	24	21.43	15	13.39	1.00
Constipation	15	13.39	9	8.04	0.05
Bitter sensation of mouth	1	0.89	0	0	1.00

Data were presented as mean ± SD.

Fisher's exact test was performed to compare the numbers of subjects with adverse effects in the two groups.
